# Using of endoscopic polypectomy in patients with diagnosed malignant colorectal polyp – The cross-sectional clinical study

**DOI:** 10.1515/med-2023-0811

**Published:** 2023-10-17

**Authors:** Vladislava Stojic, Natasa Zdravkovic, Tamara Nikolic-Turnic, Nebojsa Zdravkovic, Jelena Dimitrijevic, Aleksandra Misic, Kristijan Jovanovic, Stefan Milojevic, Jelena Zivic

**Affiliations:** Department of Medical Statistics and Informatics, Faculty of Medical Sciences, University of Kragujevac, Kragujevac, Serbia; Department of Internal Medicine, Faculty of Medical Sciences, University of Kragujevac, Kragujevac, Serbia; Department of Pharmacy, Faculty of Medical Sciences, University of Kragujevac, Kragujevac, Serbia; Department of Dentistry, Faculty of Medical Sciences, University of Kragujevac, Kragujevac, Serbia; Department of Anatomy, Faculty of Medical Sciences, University of Kragujevac, Kragujevac, Serbia; Faculty of Business Economics, EDUCONS University, Sremska Kamenica, Serbia

**Keywords:** colonic polyps, colorectal neoplasms, endoscopic polypectomy, post-polypectomy surveillance colonoscopy, surgery

## Abstract

The aim of this study was to evaluate the efficacy of endoscopic polypectomy as a therapeutic treatment for malignant alteration of colorectal polyps. In a 5-year research, 89 patients were included, who were tested and treated at the University Clinical Center Kragujevac, Kragujevac, Serbia, with the confirmed presence of malignant alteration polyps of the colon by colonoscopy, which were removed using the method of endoscopic polypectomy and confirmed by the histopathological examination of the entire polyp. After that, the same group of patients was monitored endoscopically within a certain period, controlling polypectomy locations and the occurrence of a possible remnant of the polyp, in the period of up to 2 years of polypectomy. We observed that, with an increasing size of polyps, there is also an increase in the percentage of the complexity of endoscopic resection and the appearance of remnant with histological characteristics of the invasive cancer. The highest percentage of incomplete endoscopic resection and the appearance of remnant with histological characteristics of the invasive cancer were shown at malignant altered polyps in the field of tubulovillous adenoma. Eighteen patients in total underwent the surgical intervention. In conclusion, our data support the high efficacy of endoscopic polypectomy for the removal of the altered malignant polyp.

## 1 Introduction

The removal of the malignant colon polyps during endoscopy raises a number of concerns, including the risk of the procedure and the possibility of inadequate polypectomy, ptsince these polyps have an increased risk of harboring the invasive carcinoma [1]. Colorectal cancer is most often caused by a malignant alteration in the adenoma (adenoma-carcinoma sequence) which was not promptly detected and removed (Morson theory) [2]. All adenomas have a dysplastic epithelium, which does not always take the polypoid shape and therefore the term adenoma–carcinoma sequence is replaced by the term dysplasia–carcinoma sequence [3]. In a minority of patients, colorectal cancer develops from *de novo* lesions, which grow from the mucosa [4].

The malignant potential of colorectal adenomas depends on their size, histological type, and degree of dysplasia [5]. Because of the specificity of the colorectal mucosa, unlike malignant tumors in other localizations, the malignant polyp includes intraepithelial and intramucosal carcinoma, as well as tumors, with the penetration of malignant cells into the mucosa muscularis, but not beyond [6]. The malignant polyps, respectively, represent the middle stage of the final one in the process of colorectal carcinogenesis, and the frequency of the malignant polyps is considered to be 9.5% [7]. A high-grade dysplasia (carcinoma *in situ*) and intramucosal carcinoma which contains intracryptal cell proliferation are considered to be the non-invasive cancer that has the metastatic potential. When the neoplastic tissue lesions exceed this limit, it is referred to as the invasive carcinoma [8,9].

Colorectal cancer screening and endoscopic polyp resection can reduce mortality from colorectal cancer and are now recommended by many national guidelines. Endoscopic polypectomy and endoscopic mucosal resection are the standard treatment options for colorectal neoplasia. Endoscopic submucosal dissection has great potential for the en bloc resection of larger flat or sessile lesions. However, it is technically demanding and time consuming and should be reserved for histologically advanced lesions. Endoscopic full-thickness resection is a welcome addition of endoscopic resection techniques and is very useful for the treatment of smaller difficult-to-resect lesions. The colonoscopic polypectomy technique was first described by Wolff and Shinya [10,11]. The diagnosis is made after the histological examination of entirely removed polyps [12,13,14,15]. The endoscopic polypectomy is much simpler, less expensive, and more comfortable than the surgical one; however, the final decision on the selection of the therapy depends on the shape, size, and histopathologic features of the polyp [8]. Special therapeutic problems are the malignant polyps, because of the increased possibility for the development of residual or metastatic carcinoma, especially if polypectomy was inadequate [16]. If the adenoma with the noninvasive carcinoma is completely removed with the endoscopic method, this procedure is considered curative.

There are two issues after electro-resections of colorectal polyps: whether that is the appropriate therapy for the malignant polyps, and if not, which types of polyps can lead to residues or relapse after electro-resections. That influences the decision concerning the laparotomy and resection of the intestine as a complement to adenoma electro-resections [16]. To avoid the possibility of residual or metastatic carcinoma after the endoscopic polypectomy, the subsequent endoscopy, and biopsy are required to detect possible carcinoma infiltration to the resection border area, the presence of carcinoma, and malignant affection of the lymph and blood vessels. If the malignant tissue infiltrates the border intestinal resection plates, polypectomy is considered inappropriate and the surgical resection should be performed [17].

Minimally invasive surgical techniques have proven to be superior to conventional open techniques in colorectal surgery for short-term outcomes, such as improved postoperative recovery and a reduced postoperative systemic immune response. There are various minimally invasive local excision treatments for malignant polyps, such as trans-anal resection, trans-anal endoscopic microsurgery, and laparoscopic-assisted colectomy as another minimally invasive alternative to open colorectal surgery. However, there are lack of studies that have compared the outcome of transanal minimally invasive surgery (TAMIS) and endoscopic mucosal resection.

Different complication rates between minimally invasive colorectal surgery and endoscopic polypectomy are compared in one of the studies through surgical-related outcomes and postoperative complications. Surgical-related outcomes were operating time (9.49 vs 15.28 min), blood loss (no significant differences), and lesion fragmentation rate (22.6 vs 0%), compared between the endoscopic mucosal resection and TAMIS groups. The establishment of pneumorectum and placement of a single-hole laparoscope prolong the operation time in TAMIS. The secondary outcomes were complications such as hemorrhage (higher rate in the endoscopic mucosal resection group), urinary retention (13.6% in TAMIS vs 1.9% in other groups), and postoperative infection (no significant differences) [18].

Other studies also have shown that endoscopic mucosal resection is simpler, has less morbidity and mortality, is more suitable for the treatment of rectal polyps with a longer distance from the anus, can be performed by a single person with a shorter operating time, and is cost-effective than surgery. It also should be considered the first line of treatment for patients with polyps (≥20 mm) lesions [19,20]. A meta-analysis of 50 studies, including patients with colorectal polyps treated with endoscopic mucosal resection, showed an initial success rate of 92% for endoscopic resection and 8% of patients underwent surgery due to non-curative endoscopic resection. Complications such as endoscopic recurrence, perforation, and bleeding occur in 13.8, 1.5, and 6.5% [21]. Bleeding (in 0.7–24% of the cases) and perforation (risk of 1–2%) are two main complications associated with endoscopic mucosal resection procedure [22]. Other complications include such as non-specific postprocedural pain and post-polypectomy syndrome (1%) [23].

## 2 Materials and methods

### 2.1 Ethics approval and consent to participate

The study was conducted at the Clinic of Gastroenterology and Hepatology, the University Clinical Center Kragujevac, Kragujevac, Serbia. All of the patients gave their written and informed consent to participate and the research project was approved by the Ethics Committee of the University Clinical Center Kragujevac and the Faculty of Medical Sciences, the University of Kragujevac, Serbia (n. 01/1627 and 01-311/6, date: March 8, 2010, and January 20, 2010). Additionally, adherence to the Principle of Good Clinical Practice and the Helsinki Declaration were valued throughout the process.

### 2.2 Consent for publication

All of the patients gave their consent for the publication of their data.

### 2.3 Design of study and study population

In this cross-sectional study, we investigated the total of 89 patients with the malignant altered polyp (60 male and 29 female patients; 30–89 years of age) who were, during that period of time, tested and treated at the Clinic of Gastroenterology and Hepatology, the University Clinical Center Kragujevac, Kragujevac, Serbia, and in whom colonoscopy confirmed the incidence of malignant alteration of polyps in the colon that was removed by polypectomy including endoscopic mucosal resection and confirmed by the histopathological examination of the entire polyp. The same group of patients was then followed by the endoscopic defined protocol within 2 years after the polypectomy. The study did not include the patients with initially verified existence of the invasive carcinoma through the histopathological examination of endoscopic polypectomy of the entire polyp. Neither did the study include patients diagnosed with the invasive carcinoma, after the initial polypectomy of the malignant polyps in the line of resection. Instead, these patients were sent to the surgeon. The study included patients who, besides the malignant polyps had had synchronous carcinoma at other locations that were surgically removed, and then, the malignant polyp was removed endoscopically.

### 2.4 Criteria for diagnosing the malignant altered polyp

In this study, the following described criteria were used for the diagnosis of malignant altered polyp, and histologic features that had to be included in the pathology report were emphasized.

Architectural alterations and cytologic abnormalities, principally cellular and nuclear pleomorphism, hyperchromatic cells with multilayered irregular nuclei and loss of mucin, high nuclear/cytoplasmic ratio, marked nuclear atypia with prominent nuclei, and focal cribriform patterns are considered in high-grade dysplasia. Not all these features are necessarily present to the same degree [24].

For the diagnosis of a carcinoma “*in situ*,” high dysplasia, intramucosal carcinoma or intraepithelial carcinoma, by definition, the main step is identifying changes with the above-mentioned characteristics in the stage at which they are solely confined to the epithelium, lamina propria, or muscularis mucosa and no extending into the submucosa. It is classified as pTis in the AJCC staging system and the National Comprehensive Cancer Network guidelines [25]. These terms were defined as non-invasive high-grade neoplasia in the Vienna classification [26]. Carcinoma *in situ* or severe dysplasia or intraepithelial carcinoma corresponds to a carcinoma that is restricted to the epithelial layer without invasion into the lamina propria. Intramucosal carcinoma is a carcinoma characterized by the invasion into the lamina propria. When the carcinoma spreads to the submucosa, the polyp is considered to have become malignant, being able to spread to the lymph nodes or distant sites. It is believed that the endoscopic therapy is sufficient for the malignant altered polyps that meet the following criteria (Practice Parameters Committee of the American College of Gastroenterology) [17]: the excision of the entire polyp; a regular finding of mucosa that is more than or equal to 1 mm from the edge of the polyp; well or moderately well-differentiated histology of carcinoma without lymph nodes’ invasion, without invasion of blood vessels, and a negative follow-up colonoscopy 3 months after the polypectomy.

### 2.5 Endoscopic polypectomy

Colonoscopy and endoscopic polypectomy with endoscopic mucosal resection were performed in the endoscopic cabinet of the Clinic of Gastroenterology and Hepatology, the University Clinical Center Kragujevac, Kragujevac, Serbia, using a colonoscopy device of the brand Olympus EXERA II, while the endoscopic polypectomy was performed with the electrosurgical unit of ERBE brand. The malignant polyps were removed entirely, using the standard method “in a single act” (*en bloc* resection) or “piece by piece.” The polyps from the colon were drawn along the top of the endoscope with biopic forceps or a polypectomy belt loop.

### 2.6 Histological analysis of samples

The electro-resected material was distributed and treated at the Department of Pathology of the University Clinical Center Kragujevac, Kragujevac, Serbia. The fixation was carried out in 10% formalin, the tissue was routinely processed, embedded in paraffin, and the classic method of staining with hematoxylin-eosin was applied. The histopathological examination was carried out on the entire polyps/lesions up to 3 cm in size. As for the bigger polyps, they were examined through numerous samples, to the deepest layers and the basal part of the polyp (with pedicle or without it), which corresponds to the insertion location of the polyp. The classical protocol of histopathological reports for polyps and precursor lesions contains the following information: (1) the verification and description of the received material and how representative it is; (2) the histological diagnosis and/or (sub) type of the lesion; (3) the presence and extent of neoplasia (adenoma and/or carcinoma) in samples; (4) the presence of the highest grade dysplasia or grade of histological malignancy; (5) the level of carcinoma invasion, which includes the depth and position of carcinoma; (6) the presence of vascular (lymphoid, venular) invasion; and (7) the residual status of the polypectomy: complete-radical excision of polyps (*R*_0_ category), involvement of the resection margins of a base or a pedicle (*R*_1_ category), indistinct involvement of resection margins (fragmented parts of the polyp), or estimation is not possible for other reasons (*R*_*x*_ category) [27].

### 2.7 Statistical analysis

All statistical analyses were conducted using the software package IBM SPSS Statistics version 22.0 (SPSS, Chicago, IL, USA). Categorical variables were presented in frequencies and percentages. The results were analyzed using Student’s *t*-test or a Mann–Whitney test on the dependence of normal distribution determined by a Kolmogorov–Smirnov test. For determining the correlation between the categorical variables, the chi-square test was used. The data were expressed as the mean ± standard error. All statistical analyses in this article were conducted with a confidence interval of 95%. The values of *p* < 0.05 were considered statistically significant.

## 3 Results

### 3.1 Socio-epidemiological data

Eighty-nine patients with the malignant polyp, 60 (67.4%) males and 29 (32.6%) females with the mean age of 62.5 ± 10.4 years, were studied. Two-thirds of the patients were male. The age of patients of both sexes was in the variation interval 30–89 years old. The highest frequency of patients for the whole group was in the age group 50–69 with 60.6% of the patients, and the lowest frequency was in the age group 30–49, with 13.5% of the patients. The male patients had the identical age structure as the whole group, while in the female patients, the representation was equal in the age intervals 30–49 and 70–89 years. Malignant polyps affect persons of all ages above 30, with the age frequency that increases and reaches the peak in the age group of 50–69 years. In support of this statement are *χ*² test values, with frequency schedule 30 (*p* < 0.05).

### 3.2 Characteristics of polyps and endoscopic polypectomy

All the patients had one malignant polyp each. The clinicopathological characteristics of the polyps are presented in Table 1. The dominant localization of the malignant polyps is in the sigmoid area, where the tumor was diagnosed in 77.6% of the patients. In the distal colon and rectum, 94.4% of the malignant polyps are localized, and in the right colon 5.6%. The malignant polyps were from 5 mm to over 30 mm in endoscopic size, an average of 19.2 ± 6.46. The most frequent are malignant polyps 10–19 and 20–29 mm in size that were diagnosed in 77.5% of patients – a much higher percentage in relation to the malignant polyps 1–9 and over 30 mm in size. The sigma malignant polyps are, on average, significantly bigger than the malignant polyps of other localizations. The pedunculated altered malignant polyps were 3.4 times more frequent than the polyps of the extensive base. The malignant polyps in the tubulovillous adenoma are statistically significantly more represented in relation to the malignant polyps in the tubular and villous colon adenoma (*p* < 0.05). Intramucosal depth of invasion of the malignant polyps is 2.3 times more frequent than the intraepithelial depth of invasion (*p* < 0.05).

**Table 1:** Epidemiological data, polyps’ characteristics, and endoscopic polypectomy

**Number of patients**	**89**
**Age (years), mean (s.d.)**	62.5 ± 10.4
**Gender,** ***n*** **(%)**	
Women	29 (32.6%)
Men	60 (67.4%)
**Location,** ***n*** **(%)**	
Rectum	13 (14.6%)
Sigmoid colon	69 (77.6%)
Descending colon	2 (2.2%)
Transverse colon	2 (2.2%)
Ascending colon	2 (2.2%)
Cecum	1 (1.1%)
**Size,** ***n*** **(%)**	
1–9 mm	8 (8.9%)
10–19 mm	38 (42.7%)
20–29 mm	31 (34.8%)
>30 mm	12 (13.5%)
**Configurations,** ***n*** **(%)**	
Pedunculated	65 (73.1%)
Sessile	22 (24.7%)
Flat	2 (2.2%)
**Histological characteristics,** ***n*** **(%)**	
Tubular adenomas	23 (25.8%)
Villous adenomas	18 (20.2%)
Tubulovillous adenomas	48 (53.9%)
Polypectomy, *n* (%)	
Complete resection	62 (69.7%)
Incomplete resection	27 (30.3%)
**Technique of polypectomy,** ***n*** **(%)**	
In one act	71 (79.8%)
Piece by piece	18 (20.2%)
**Depth of invasion,** ***n*** **(%)**	
Intraepithelial	27 (30.3%)
Intramucous	62 (69.7%)
**Line of resection,** ***n*** **(%)**	
Not available	20 (22.5%)
Without dysplasia	47 (52.7%)
Dysplasia grade I	2 (2.3%)
Dysplasia grade II	3 (3.4%)
Dysplasia grade III	8 (9.0%)
Carcinoma *in situ*	9 (10.1%)
**Residual status,** ***n*** **(%)**	
Radical excision of polyps	47 (52.8%)
Vague involvement of resection margins	37 (41.6%)
Involvement of resection margins	5 (5.5%)

At localities of the malignant polyps, polypectomy in 69.7% of patients who had endoscopic controls at prescribed intervals within 2 years, the control colonoscopy did not verify the residual adenomatous tissue, recurrence of polyps, nor nodular growth. In 30.3% of patients, polypectomy was incomplete. In 79.8% of total study patients, polypectomy was performed in one act, and in 52.7% of patients, after polypectomy, the line of resection with no signs of dysplasia and invasion was verified. Radical excision of polyp was diagnosed in 52.8% of patients.

### 3.3 Histopathological characteristics of the polypectomy localization

In the group of patients with incomplete resection, 11 patients were diagnosed with benign remnant histopathological characteristics, and in 16 patients, the remnant was with malignant histological characteristics. All 11 patients with benign remnant histopathologic features had the endoscopic treatment and had a regular finding at the location of previous polypectomy in endoscopic examinations within 2 years of the follow-up period (Table 2).

**Table 2:** Histological characteristics of the polypectomy localization

Histological characteristics	Percentage (%)	Number
Tubular adenomas	22.2	6
Villous adenomas	7.4	2
Tubulovillous ademonas	11.1	3
Invasive carcinoma	29.7	8
Carcinoma *in situ*	29.6	8

**Figure 1:**
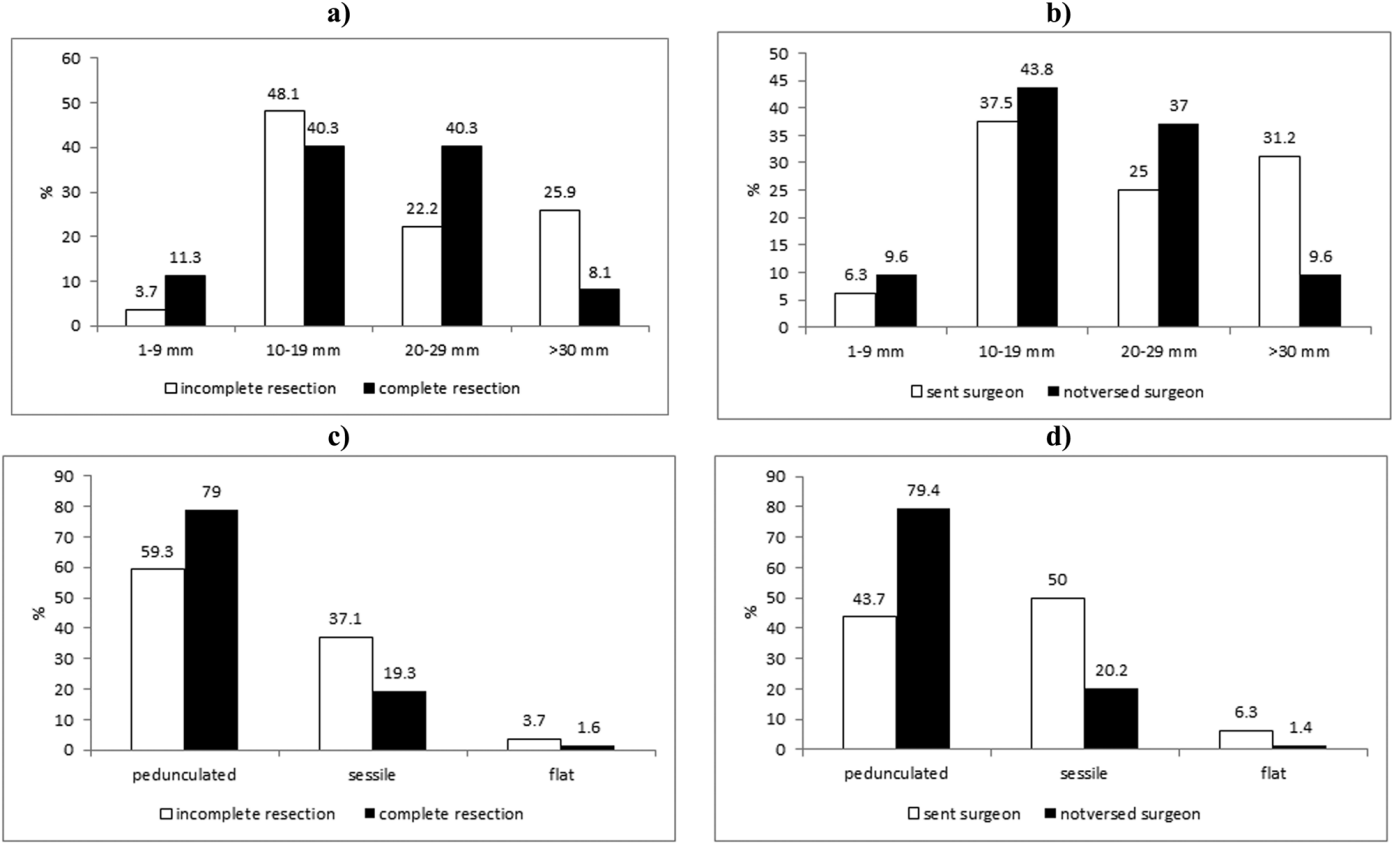
Incomplete resection and referral to the surgeon according to the size and configuration of the polyp. (a) With an increase in the size of polyps, there grows also the percentage of the incomplete resection. (b) The highest percentage of referral to the surgeon is in the category of polyps size over 30 mm. (c) Higher percentage of incomplete resection in the sessile configuration of the malignant polyps. (d) Patients with sessile configuration of malignant-altered polyps were referred to the surgeon.

The lowest percentage of incomplete resection was identified in the malignant polyps 1–9 mm in size. With an increased size of polyps, there is also an increase in percentage of the incomplete resection, so that it is 3.2 times more frequent in a group of the malignant polyps bigger than 30 mm (*p* < 0.05) (Figure 1a).

The examination of the number of patients who were referred to the surgeon and those who were not, for each size category of polyps, revealed that the group of polyps larger than 30 mm had the highest percentage of referrals to the surgeon (*χ*² = 0.036, *p* < 0.05) (Figure 1b).

A higher percentage of incomplete resection was verified in a sessile configuration of the malignant polyps (*p* < 0.05) compared to other macro-types (Figure 1c). The patients with sessile configuration of the malignant polyps were 2.8 times more referred to the surgeon, compared to the patients with a peduncle configuration (*χ*² = 0.008, *p* < 0.05) (Figure 1d).

**Figure 2:**
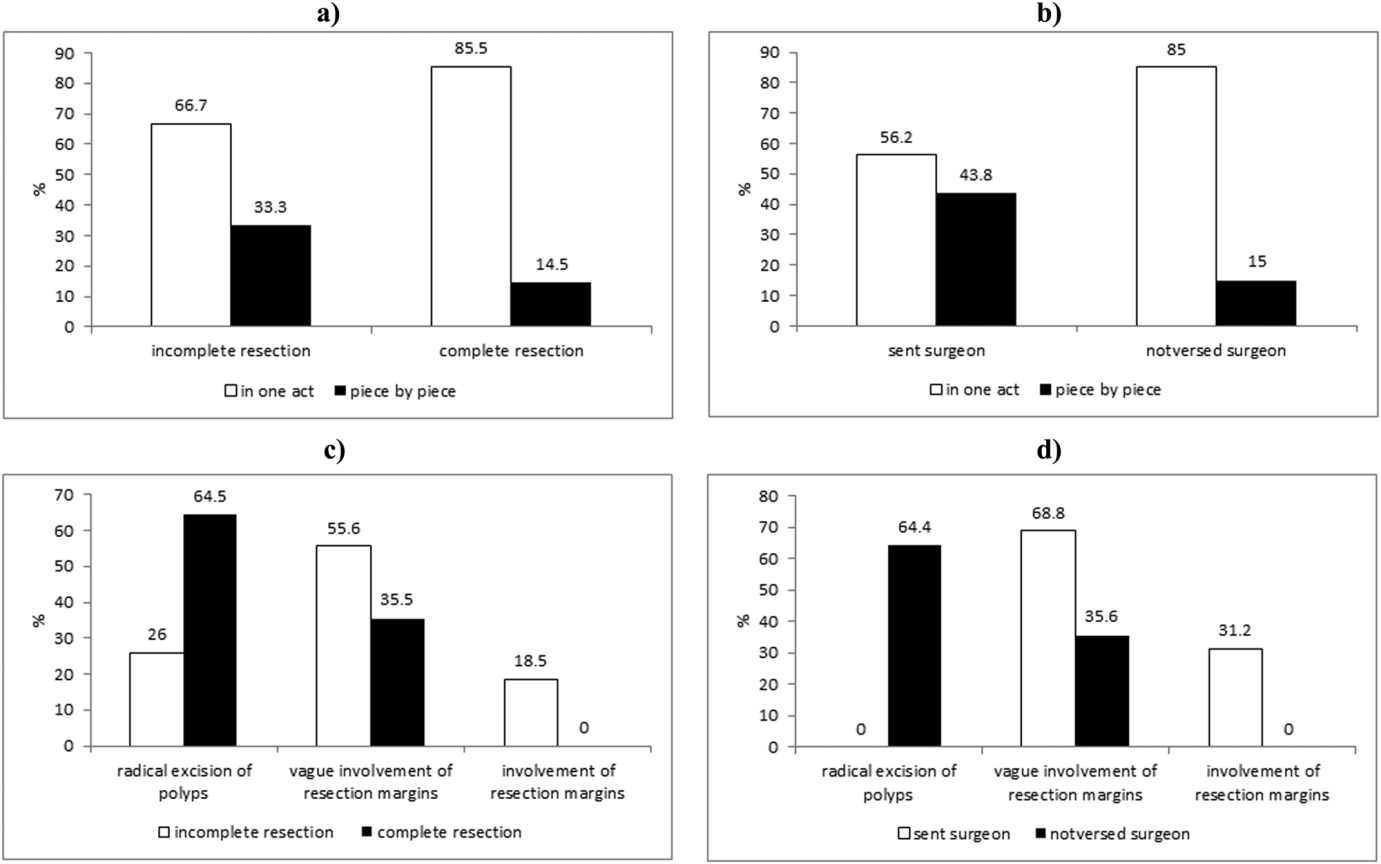
Incomplete resection and referral to the surgeon according to the type of polypectomy and residual polypectomy status. (a) Higher percentage of incomplete resection is in polypectomy technique piece by piece. (b) The percentage of referral to the surgeon is greater in those patients with the malignant polyps, in which resection was performed using technique piece by piece. (c) Verified incomplete polypectomy in 7 patients with the radical excision of polyps, and in 5 patients including involvement of resection margins. (d) Surgeon is not addressed to a single patient with a residual status that showed the radical excision of polyp.

In patients with incomplete resection of the malignant polyps, it was 66.7% who had polypectomy in one act and 33.3% who had polypectomy performed in a technique piece by piece. A higher percentage of incomplete resection is seen in a polypectomy technique piece by piece (*p* < 0.05) (Figure 2a).

In the group of 16 patients who were referred to the surgeon, 56.2% polypectomy was performed the “in one act” technique and 43.8% the “piece by piece” technique. The percentage of referral to the surgeon is greater in those patients with malignant-altered polyps, in which resection was performed using the “piece by piece” technique (*χ*² = 0.016, *p* < 0.05) (Figure 2b).

### 3.4 Distribution of polypectomy localizations in relation to the histopathological alterations

**Figure 3:**
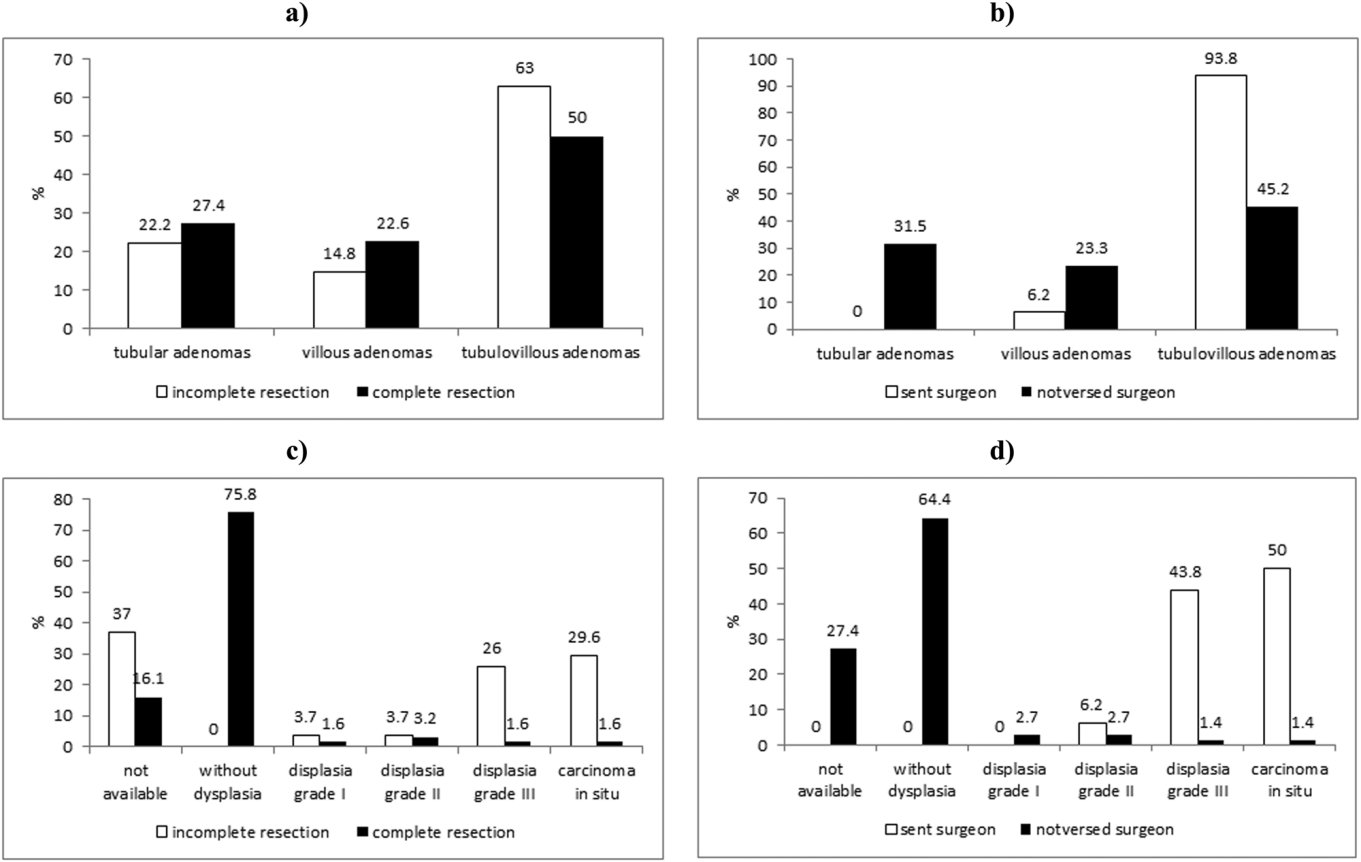
Incomplete resection and referral to the surgeon according to the histopathological characteristics and resection line. (a) Incomplete resection is more common in the tubulovillous malignant polyps. (b) The highest percentage of patients with the malignant-altered tubulovillous polyp was referred to the surgeon. (c) A high percentage of incomplete resection has been verified in resected polyp dysplasia grade III and a focus of carcinoma *in situ* at the line of resection. (d) With the gradation lines, resection increases the percentage of patients referred to the surgeon.

Analyzing the polypectomy localizations, depending on the histopathological characteristics of the malignant polyps, we got the results that in the group of patients with incomplete resection, most of the malignant polyps were in the area of tubulovillous adenoma, however, without statistical significance (Figure 3a). The surgeon has referred 15 patients with malignant changed tubulovillous adenoma. No patient with malignant changed villous adenoma was identified (*χ*² = 0.043, *p* < 0.05) (Figure 3b).

Incomplete resection was verified at 22.2% of malignant-altered polyps with intraepithelial depth of invasion, and 77.8% with intramucosal depth of invasion. The completeness of resection is independent of the depth of invasion of the malignant-altered polyp which is removed by polypectomy (*p* > 0.05). The patients referred to the surgeon scaled as 31.3% of patients with intraepithelial depth of invasion of the malignant polyps and 68.7% with intramucosal depth of invasion of the malignant polyps. The percentage of patients referred to the surgeon does not depend on the depth of invasion of malignant-altered polyps (*χ*² = 0.754, *p* > 0.05).

### 3.5 Distribution of malignant polyps with/without resection

All malignant polyps with a resection line with no signs of dysplasia and invasion were fully resected. A high percentage of incomplete resection was verified in resected polyp dysplasia grade III with a focus of carcinoma *in situ* at the line of resection (Figure 3c). The completeness of resection is dependent on the line of resection (*χ*² = 0.039, *p* < 0.05), and odds ratio is 4.928 (each gradation line of resection of increased risk for restoration to about 2 times). With the gradation of the line of resection, the percentage of patients referred to the surgeon increases, so that no patient was referred to the surgeon with the line of resection without signs of dysplasia and the invasion, nor with dysplasia grade I. One patient who was sent to the surgeon had the line of resection of a dysplasia grade II and 7 patients referred to the surgeon had the line of resection grade III dysplasia. Eight patients referred to the surgeon had on the line of resection with the focus of carcinoma *in situ* (Figure 3d).

The frequency of referring to the surgeon depends on the line of resection (*χ*² = 0.009, *p* < 0.05), and the odds ratio is 3.441 (each gradation of the line of resection increases the risk of referring to the surgeon about 3.5×). Incomplete polypectomy was verified in 7 patients with a radical excision of polyps, and in 5 patients including involvement of resection margins (*p* < 0.05) (Figure 2c). 68.8% of patients with an unclear margin were sent to the surgeon as well as 31.2% of patients with a clear one including involvement of resection (*χ*² = 0.000, *p* < 0.05) (Figure 2d). No patient with a residual status showing the radical excision of polyp (*χ*² = 0.000, *p* < 0.05) was referred to the surgeon.

### 3.6 Histopathological features of polypectomy *in situ* referred to the surgeon

The clinicopathological features of *in situ* polypectomy referred to the surgeon are presented in Table 3. Eight out of 16 patients who went to the surgeon’s check-up after the endoscopic examination had carcinoma *in situ* at the polypectomy location, 7 invasive carcinomas, and one of them, dysplasia grade III. The highest percentage of patients with the remnant of invasive cancer had the sigmoid localization of malignant-altered polyps (*p* = 0.001), over 30 mm in size (*p* = 0.001), of the sessile configurations (*χ*² = 0.024, *p* = 0.001, odds ratio 4.957) at the area of tubulovillous adenoma (*p* = 0.001). With each gradation of the line of resection, the risk of malignant remnant characteristics increases by 5 times (*χ*² = 0.010, *p* < 0.001, odds ratio 5.041). There was no remnant with a malignant histological status, after the radical excision of the malignant-altered polyp; in 45.5% of the patients with unclear invasion of the resection margins, there was rest and histological characteristics of the invasive carcinoma and the same percentage is obtained in the group where invasion of the resection margins was confirmed (*χ*² = 0.001, *p* < 0.001).

**Table 3:** Clinicopathological features of the polypectomy localization addresed surgeon

	Dysplasia grade III	Carcinoma *in situ*	Invasive carcinoma	*p* value
**Location,** ***n*** **(%)**				
Rectum	0 (0.0%)	3 (75.0%)	1 (25%)	
Sigmoid colon	1 (9.0%)	3 (27.2%)	7 (63.6%)	*p* = 0.001
Descending colon	0 (0.0%)	0 (0.0%)	0 (0.0%)	
Transverse colon	0 (0.0%)	1 (100.0%)	0 (0.0%)	
Ascending colon	0 (0.0%)	0 (0.0%)	0 (0.0%)	
Cecum	0 (0.0%)	0 (0.0%)	0 (0.0%)	
**Size,** ***n*** **(%)**				
1–9 mm	0 (0.0%)	1(100.0%)	0 (0.0%)	
10–19 mm	1 (16.6%)	3 (50.0%)	2 (33.3%)	
20–29 mm	0 (0.0%)	3 (75.0%)	1 (25.0%)	
>30 mm	0 (0.0%)	1 (20.0%)	4 (80.0%)	*p* = 0.005
**Configurations,** ***n*** **(%)**				
Pedunculated	0 (0.0%)	3 (57.1%)	3 (42.9%)	
Sessile	1 (12.5%)	3 (37.5%)	4 (50.0%)	*p* = 0.042
Flat	0 (0.0%)	1 (100.0%)	0 (0.0%)	
**Histological characteristics,** ***n*** **(%)**				
Tubular adenomas	0 (0.0%)	0 (0.0%)	0 (0.0%)	
Villous adenomas	1 (100.0%)	0 (0.0%)	0 (0.0%)	
Tubulovillous adenomas	0 (0.0%)	8 (53.3%)	7 (46.7%)	*p* = 0.003
**Technique of polypectomy,** ***n*** **(%)**				
In one act	1 (11.1%)	7 (77.8%)	1 (11.1%)	
Piece by piece	0 (0.0%)	1 (14.3%)	6 (85.7%)	*p* = 0.023
**Depth of invasion,** ***n*** **(%)**				
Intraepithelial	1 (20.0%)	3 (60.0%)	1 (20.0%)	
Intramucous	0 (0.0%)	5 (45.5%)	6 (54.5%)	
**Line of resection,** ***n*** **(%)**				
Not available	0 (0.0%)	0 (0.0%)	0 (0.0%)	
Dysplasia grade I	0 (0.0%)	0 (0.0%)	0 (0.0%)	
Dysplasia grade II	0 (0.0%)	1 (100.0%)	0 (0.0%)	
Dysplasia grade III	1 (14.3%)	2 (28.6%)	4 (56.1%)	
Carcinoma *in situ*	0 (0.0%)	5 (62.5%)	3 (37.3%)	*p* = 0.001
**Residual status,** ***n*** **(%)**				
Radical excision of polyps	0 (0.0%)	0 (0.0%)	0 (0.0%)	
Vague involvement of resection margins	1 (9.1%)	5 (45.4%)	5 (45.4%)	
Involvement of resection margins	0 (0.0%)	3 (60.0%)	2 (40.0%)	*p* = 0.003

## 4 Discussion

The progress of the visual techniques [28,29,30] and techniques of polypectomy, which allow detection and removal of polypoid transformations, have resulted in a decrease in the incidence of colorectal carcinoma [31,32,33], which is consistent with the theory of adenoma-carcinoma sequence [34,35]. The precursor lesions for developing the colorectal cancer have been defined, as well as the aberrant crypt focuses [36,37,38]; the genes responsible for colorectal apoptosis have been discovered [39,40,41], as well as the genes responsible for the incidence of familial adenomatous polyposis [42], and hereditary un-polypoid colorectal cancer [43,44,45,46]. Flat adenomas [47,48,49,50] and a flat colorectal carcinoma [30,51], adenomas showing a depressed surface, colorectal carcinoma [52,53] as well as de novo colorectal carcinoma [54] have been described in the literature so far.

However, in spite of those important discoveries and satisfactory doctrine of primary and secondary preventions of colorectal carcinomas, the incidence is increasing. Good prevention of colorectal carcinomas implies timely diagnosis and removal of adenomas. It is necessary to make clinical, endoscopic, histologic, and histochemical analyses to define the optimal plan for further treatment. The removal of the malignant polyps directly prevents cancer development and reduces the incidence of colorectal cancer to 90.0% [55].

Based on the number of colonoscopy examinations, the number of patients who had polypectomy, and the total number of endoscopically removed polyps in our study, the malignant polyps were diagnosed in 12.5% of patients who had polypectomy and represent 8.7% of all polyps removed endoscopically. The literature data show that the malignant polyps are diagnosed in 0.2–9.0% of endoscopically removed adenomatous polyps, and 9.0–11.0% of the surgically treated polyps [51]. The diagnosis of malignant alteration of the adenoma is exclusively histological. Friability, hardness, ulceration, the shape of “Christmas tree,” the asymmetry [52], and to a lesser extent, lobular surface adenoma are macroscopic criteria indicating malignancy. Theoretically, the endoscopic polypectomy is curative; however, in practice, there are some restrictions [53]. Recommendations of some authors have clearly defined that adenomas with carcinoma *in situ* should be treated only by polypectomy [51,52,53].

In our study, at polypectomy localizations of the malignant polyps in 69.7% of patients, who were endoscopically controlled at prescribed intervals up to 2 years, the control colonoscopy did not verify the residual adenomatous tissue, recurrence of polyps, nor nodular growth. In 30.3% of patients, the control colonoscopy verified relapse. In the group of patients with incomplete resection, 11 patients were diagnosed with residual adenomatous tissue benign pathohistological characteristics and 16 patients had malignant histological characteristics. All 11 patients with benign pathohistological characteristics were resolved endoscopically and they had neat polypectomy sites at control endoscopic examinations in follow-up 2 years later. A high percentage of success of the polypectomy of the malignant polyps with normal endoscopy findings at control colonoscopy examinations is described also by other authors [34,35,36,37,38,39,40].

Also, in our study, the highest frequency of patients for the group as a whole is at the age group 50–69 years. The prevalence of malignant polyps was in a decline after the age of 70, which is explained by the fact that the small number of patients of this age had been examined, due to intolerance to the examination or reduced possibilities of patients of that age to be correctly prepared for colonoscopy. According to Morson and associates [53] the malignant polyps most frequently occur at the age of 60–79 years. The slightly higher average age of the patients was verified in studies by Seitz and associates [55]. The older age is a risk factor, because during aging, there is an accumulation of genetic changes and mutations in the cells. Hakama et al. noted the risk for developing colorectal cancer considering the age and displayed the rate of incidence of 19.2/100,000 in patients older than 65 and 37.1/100,000 in patients older than 65 [54].

We have shown sexual predilection, i.e., a dominant representation of the malignant polyps in male patients. Song et al. [53] showed some higher prevalence of the malignant polyps in females. In some reports, there is an equal representation of both sexes [55]. The explanation for the evident difference between the sexes can be found in the results of controlled experiments. It has been proven that men and women differ in terms of the intestinal transit time, volume of feces, and production of short-chain fatty acids and bile acids [48,51,53].

The malignant polyps in the colon can be found in all of its segments. The dominant localization of the malignant polyp is in the sigmoid area. There is an evident trend of decreasing the prevalence of malignant polyps from distal toward proximal segments. The higher appearance of the malignant polyps distally located is confirmed by other authors [34,41,52,54]. The observed higher incidence of distal parts, verified in our and other studies, explains the earlier manifestation of symptoms in the distal colon compared to the proximal. There are studies, in which the results indicate a trend of increase in the incidence of colorectal carcinoma in the proximal segment of the large intestine, instead of a decrease in the distal parts [41].

There are suggested different genetic mechanisms of cancer appearance in the right and left colon and rectum. It is believed that the increase in the incidence of colorectal carcinoma in the right column is a result of the routine use of colonoscopy [36]. The most common localization of the malignant polyps at the distal part of the colon is in accordance with the predominant prevalence of colorectal cancer in precisely this part of the colon, which is one of the significant confirmations of the theory of adenoma-carcinoma sequence. The altered malignant polyps, regardless of the histological structure, were most rarely represented in the caecoascedent part of the colon. Since malignant polyps may be found in all segments of the colon, colonoscopy is the method of choice for the optimal endoscopic analysis of such tumors.

The most common are malignant polyps 10–29 mm in size. It has been observed that the increasing size of the malignant polyp is followed by the trend of malignant polyp localization in the distal segments of the colon. Many studies [53,55] have revealed an increasing incidence of the malignant polyp size >20 mm. The increase in the size of the adenoma increased the risk for malignancy [31,37,53,56]. Some studies have shown that a *K*-ras mutation is more frequently represented in larger adenomas [38].

The macroscopic tumor type is an important factor in determining the recurrence and metastatic potential of tumor cells [30]. By analyzing the configuration of the polyp, we have shown the dominance of the peduncular polyps, which are 3.4 times stronger than the polyp of the extensive base. Similar results have been shown in studies by other authors [56,57,58,59].

Significantly more frequent incomplete resection of the polyp was at those sessile configurations and patients with sessile configurations of malignant-altered polyps were 2.8 times more often referred to the surgeon. Similar results were shown by other authors [52]. The sessile macro-morphological configuration of the adenoma facilitates the development of residual and metastatic cancer. In all these cases, surgical intervention is necessary [27]. A polyp with a non-invasive carcinoma becomes an invasive polyp in a time [42] which can be interrupted by a polypectomy [37].

Endoscopic polypectomy and endoscopic mucosal resection were the endoscopic techniques available in our study for the removal of colon polyp. Considering endoscopic mucosal resection as a lower risk of adverse events, relatively simple to perform, and endoscopic submucosal dissection as a more complex, high-risk procedure performed by endoscopists associated with a higher perforation rate.

The most common complication after the endoscopic mucosal resection is bleeding, reported in 0.7–24% of the cases. Intraprocedural bleeding has been reported in 11–22% of cases [60].

The risk factor for intraprocedural bleeding includes large polyps, tubulovillous or villous lesion, and minimally elevated sessile polyps. Previously published articles showed that postprocedural bleeding occurs in 2–11% of cases, however, the rate of clinically significant bleeding is present in only 6% of cases and occurs hours to days after the procedure [61]. The bleeding rate after submucosal endoscopic resection has been reported in 0–11.9% for up to 15 days post-procedure [61]. Meta-analysis of 104 studies showed the rate of immediate and delayed major bleeding after submucosal endoscopic resection for colorectal lesions of 0.75 and 2.1% [62]. Risk factors for delayed bleeding include the lesion’s size, sessile type, the occurrence of intraprocedural bleeding, use of prior anti-thrombotic agents, and lesions in the cecum and rectum with a higher incidence [63].

Perforation is also a potential complication after endoscopic mucosal and submucosal resections. The perforation rate after endoscopic mucosal resection is low, reported as 1–2%.

Perforation is more common following colorectal endoscopic submucosal resection, with reported rate 3.3–10% [64]. Risk factors include using larger diameter snares (≥20 mm), proximal location, bulky lesions, and cutting current. Meta-analysis of 66 studies comparing these two endoscopic procedures for colorectal lesions found higher perforation rate with submucosal compared to mucosal resection [65]. Risk factors for perforations during submucosal resection, besides tumor size and location, include submucosal fibrosis and perforations are more in the ascending colon and cecum due to its thin wall. With reference to 2022 European Society of Gastrointestinal Endoscopy (ESGE) guidelines, submucosal endoscopic resection should be considered for *en bloc* resection of colorectal (but particularly rectal) lesions with suspicion of limited submucosal invasion (demarcated depressed area with irregular surface pattern or a large protruding or bulky component, particularly if the lesions are larger than 20 mm) or for lesions that otherwise cannot be completely removed by snare-based techniques [66]. Submucosal endoscopic resection showed benefits in the technical, histological, and oncological outcomes as it provides curative treatment without the need for surgery for lesions with a significant likelihood of submucosal invasion. ESD showed benefits in the technical, histological, and oncological outcomes as it provides curative treatment, associated with higher rate of *en bloc* and complete resection and lower recurrence compared to mucosal resection but, at the cost of increased procedural time, needs for additional surgical operations and perforation risk. [67].

Most of the malignant polyps are removed by polypectomy in one act; however, a higher percentage of incomplete resections was verified using the technique piece by piece. The results of other studies also show a slightly higher percentage of polypectomy technique in one act [43,44,45,46,47,52].

Most of the malignant polyps, as well as the highest percentage of remnant polyps, were in the area of tubulovillous adenoma type. The reports of other studies have shown the highest percentage of the polyp in the field of tubular adenoma, and the highest percentage of malignant alteration is in the field of villous adenoma, wherein the percentage of malignant tubulovillous adenomas is closer to the villous type instead of the tubular adenoma types [52,53,54,55,56,57]. The data from these and other studies are confirmation that the villous adenomas are the rarest histologic type of adenomas of the colon, but with the greatest malignant potential. Despite numerous tests, it cannot be claimed with certainty that the histological structure of villous adenoma is only directly responsible for the increased level of their malignant alteration.

The line of resection on the site of malignant-altered polyps which were removed by polypectomy in patients with complete resection was, in the highest percentage, without dysplasia and invasion. The radical excision of the polyp existed in 52.8% of patients and none of the patients from this group were sent to the surgeon. Five patients were diagnosed with the clear resection margin involvement, and all five patients on the control endoscopy had histological characteristics of remnant carcinoma *in situ* and invasive carcinoma and all were sent to the surgeon. The recurrent potential of adenoma or adenoma with carcinoma depends on the residual status, hereditary load, age, anatomical location, and histopathological features [56,58].

The group of patients who were referred to the surgeon for further treatment had shown malignant polyps over 30 mm in size and sessile configurations in the field of tubulovillous adenoma with a clear and ambiguous invasion of resection margins. The efficiency of a polypectomy and the rest of sessile polyps are dependent on several factors: the size of the polyp, the resection technique, endoscopist experiences, and histological type [54].

It should be noted that all adenomas grow over time and can change their size and the level of their appeared components. The process of malignant transformation is a lengthy multistep process that, depending on the characteristics of the adenoma, can last for years. In terms of the residual status, it is shown that for *R*_0_ status, there is a high risk of recurrence or alterations and it requires an intensive monitoring within 3, 6, and 12 months. *R*_*x*_ and *R*_1_ are the status of a very high risk of recurrence or alteration with an intense monitoring or surgical resection [16]. The presence of neoplasia/cancer in adenoma always gives a long-term risk of adenoma recurrence (20.0–60.0% in the first 2 years) or the occurrence of carcinoma in the colon (5.0% in the next 15 years). The metastatic risk of the malignant polyps is dependent on the existence of submucosal invasion, presence of lymphatic-vascular invasion in 9.7–44.0% of neoplastic lesions, venular invasion in 3.5% malignant polyps, residual tumors at the resection margins, and poor histological differentiation of carcinoma [31,58,59].

Witold and colleagues have tried to define the histopathological criteria that were used to decide the therapeutic treatment of the malignant polyps [59]. The distance of the tumor from the resection margin, which is less than 1 mm of resection and/or grade III and/or lymphatic invasion and/or venous invasion, has been defined as unfavorable histological criteria [59]. The post-polypectomy follow-up period confirmed that the malignant polyp size over 3 cm with the incomplete resection, vascular space invasion, and poor histological differentiation requires the surgical resection [58].

## 5 Conclusions

Our data support the high efficacy of endoscopic polypectomy for the removal of the malignant polyp. Histology of the varied polyps and polypectomy were an adequate treatment except in cases with an invasive cancer. Most polyps were located in the left colon. The endoscopic polypectomy is effective in the removal of the malignant polyp and, thus, it reduces the risk of developing cancer, as supported by the literature. A regular follow-up program for these patients is mandatory.
